# The health-related social costs of alcohol in Belgium

**DOI:** 10.1186/s12889-017-4974-4

**Published:** 2017-12-16

**Authors:** Nick Verhaeghe, Delfine Lievens, Lieven Annemans, Freya Vander Laenen, Koen Putman

**Affiliations:** 1 Department of Public Health, Interuniversity Centre for Health Economics Research (I-CHER), Vrije Universiteit Brussel, B-1090 Jette, Belgium; 2 Department of Public Health, Interuniversity Centre for Health Economics Research (I-CHER) , Ghent University, B-9000 Ghent, Belgium; 3 Institute for International Research on Criminal Policy (IRCP), Ghent University, B-9000 Ghent, Belgium

**Keywords:** Alcohol, Cost-of-illness, Health, Belgium

## Abstract

**Background:**

Alcohol is associated with adverse health effects causing a considerable economic impact to society. A reliable estimate of this economic impact for Belgium is lacking. This is the aim of the study.

**Methods:**

A prevalence-based approach estimating the direct, indirect and intangible costs for the year 2012 was used. Attributional fractions for a series of health effects were derived from literature. The human capital approach was used to estimate indirect costs, while the concept of disability-adjusted life years was used to estimate intangible costs. Sensitivity and scenario analyses were conducted to assess the uncertainty around cost estimates and to evaluate the impact of alternative modelling assumptions.

**Results:**

In 2012, total alcohol-attributable direct costs were estimated at €906.1 million, of which the majority were due to hospitalization (€743.7 million, 82%). The indirect costs amounted to €642.6 million, of which 62% was caused by premature mortality. Alcohol was responsible for 157,500 disability-adjusted life years representing €6.3 billion intangible costs.

**Conclusions:**

Despite a number of limitations intrinsic to this kind of research, the study can be considered as the most comprehensive analysis thus far of the health-related social costs of alcohol in Belgium.

## Background

Alcohol is a worldwide problem affecting the health and economic welfare of societies due to a number of adverse events. Every year, alcohol is responsible for 3.3 million deaths (5.9% of all deaths worldwide) and 5.1% of the global burden of disease [1]. It increases the risk of developing a number of diseases including liver cirrhosis, neuropsychiatric disorders, cancers, circulatory diseases, and injuries such as road traffic accidents and falls [2]. By the negative impact on health, alcohol has a considerable effect on health care services’ use, productivity, and health-related quality of life (HRQOL), and consequently on costs [2, 3].

Given the substantial economic impact of alcohol to society, insights into the magnitude of its costs are important. The economic burden of alcohol to society can be estimated by cost-of-illness studies [4]. In such studies, diseases’ social costs are measured by estimating the direct, indirect, and intangible costs. Direct costs are those to deal with the alcohol (mis)use or its proximate effects (for example hospitalisation, nursing care, medication). Indirect costs are those related to lost human productivity due to alcohol (for example productivity losses due to morbidity or mortality). Intangible costs can be considered as non-financial welfare losses such as reduced HRQOL [4, 5]. The guiding principle in a cost-of-illness framework is that a social problem imposes costs when resources are used as a result of that problem, whereas they could have been used differently [4]. The costs due to the substance in a societies’ current situation are compared with a counterfactual situation, usually the more or less unreal scenario of no existence of the substance [6]. Several studies already addressed the economic burden of alcohol to society. In Scotland in 2002, alcohol-attributable healthcare costs were estimated to be £95.6 million, while indirect costs amounted to £404.5 million [7]. In Germany in 2002, alcohol-attributable direct and indirect costs amounted to €24.4 million [8]. In Sweden in 2002, direct and indirect alcohol-attributable costs were found to be 29.4 million Swedish kronor [€3.0 million]. In this study, crime costs were also included [9]. Comparing these findings is yet difficult due to variations in cost items included across the studies. For example, in the Scottish study [7], alcohol-attributable costs related to Accident and Emergency Care and unemployment were included, while they were not in the German [8] and Swedish study [9]. In Belgium, alcohol-attributable costs have also been studied. In the study by Vander Laenen et al. [10], the cost estimates were limited to the direct expenditures by public authorities including expenditures on prevention, treatment, harm reduction, and security. For the reference year 2008, total costs amounted to €627 million. In another Belgian study [11], direct and indirect costs were estimated, however omitting the intangible costs. It is clear that the economic impact of alcohol to the Belgian society is more far-reaching than what has been estimated by those studies. Contrary to the previous Belgian studies where only specific elements of the alcohol-attributable social costs were estimated, the aim of the current study was to perform a more comprehensive analysis estimating the health-related social costs including direct, indirect, and intangible cost categories of alcohol in Belgium.

## Methods

A prevalence-based approach was used measuring the consequences of alcohol in Belgium for the year 2012. A prevalence-based study estimates the economic burden of a disease or condition over a specific period, typically a year [5]. Similar to the study by Jarl et al. [9], we considered all alcohol consumption levels, and not only alcohol misuse. The counterfactual scenario was defined as alcohol use causing no costs to society. Diseases (based on ICD-9 diagnosis) known to be causally related to alcohol use were identified using the ‘International guidelines for estimating the costs of substance abuse – 2003 Edition’ [12]. In addition, published social costs studies were searched to identify diseases not included in the guidelines (for example unipolar major depression) [13, 14]. A number of diseases are by definition fully attributable to alcohol including ‘alcoholic psychosis’ (ICD-9 code 291), ‘alcoholic dependence syndrome’ (303), ‘alcohol abuse’ (305), ‘degeneration of nervous system due to alcohol’ (331.7), ‘alcoholic polyneuropathy’ (357.5), ‘alcoholic cardiomyopathy’ (425.5), ‘alcoholic gastritis’ (535.3), ‘alcoholic liver disease’ (571), and ‘foetal alcohol syndrome’ (760.71). Others are partially associated with alcohol and for those the epidemiological concept of alcohol-attributable fractions (AAF) [15] was used to quantify the number of cases of diseases and deaths that could be attributed to alcohol. AAFs are calculated as follows:

$$ \mathrm{AAF}={\sum}_{\mathrm{i}=1}{\mathrm{P}}_{\mathrm{i}}\left({\mathrm{RR}}_{\mathrm{i}}\hbox{-} 1\right)/{\sum}_{\mathrm{i}=0}{\mathrm{P}}_{\mathrm{i}}\left({\mathrm{RR}}_{\mathrm{i}}\hbox{-} 1\right)+1 $$

where P_i_ signifies the prevalence of alcohol consumption in consumption class i and RR_i_ signifies the relative risk of disease in alcohol consumption class i. Alcohol consumption data were obtained from the publicly available online ‘interactive analysis’ tool from the ‘Belgian Health Interview Survey 2013’ [16]. Four age- and sex-specific drinking classes were considered (Table 1). Relative risk data were obtained from previous studies (Table 2). An overview of the calculated age- and sex-specific AAFs can be found in Table 3.

**Table 1:** Age- and sex-specific alcohol consumption (%) per drinking category in Belgium, 2012

Age-band (years)	Males (%)				Females (%)			
	Abstinent	Cat. I	Cat. II	Cat. III	Abstinent	Cat. I	Cat. II	Cat. III
<19	26.4	71.0	1.3	1.3	22.3	75.3	1.8	0.6
20–39	12.9	84.5	1.3	1.3	22.4	75.6	1.5	0.5
40–59	10.7	82.5	3.3	3.5	16.6	76.1	5.5	1.8
60–79	12.8	79.4	3.6	4.2	24.1	70.9	4.0	1.0
≥80	28.6	69.9	0.7	0.8	47.4	49.6	2.4	0.6

Males: cat. I, 0–39.9 g/day; cat. II, 40–59.9 g/day; cat. III, ≥60 g/day

Females: cat. I, 0–19.9 g/day; cat. II, 20–39.9 g/day; cat. III, ≥40 g/day

**Table 2:** Relative risks for diseases partially associated with alcohol use by sex and drinking category

Disease	Males			Females			Source
	Cat. I	Cat. II	Cat. III	Cat. I	Cat. II	Cat. III	
Lip, oral cavity, pharynx cancer	1.45	1.85	5.39	1.45	1.85	5.39	[43]
Oesophageal cancer	1.80	2.38	4.36	1.80	2.38	4.36	[43]
Rectal cancer	1.08	1.30	1.72	NA	1.11	1.33	[44]
Liver cancer	1.45	3.03	3.60	1.45	3.03	3.60	[43]
Pancreatic cancer	1.10	1.30	1.70	1.10	1.30	1.70	[45]
Laryngeal cancer	1.83	3.90	4.93	1.83	3.90	4.93	[43]
Breast cancer <45 year	NA	NA	NA	1.15	1.41	1.46	[43]
Breast cancer ≥45 year	NA	NA	NA	1.14	1.38	1.62	[43]
Unipolar major depression	1.19	2.49	2.12	1.66	3.98	4.32	[46]
Epilepsy	1.23	7.52	6.83	1.34	7.22	7.52	[43]
Hypertension	1.40	2.00	4.10	1.40	2.00	2.00	[43]
Cardiac dysrhythmia	1.51	2.23	2.23	1.51	2.23	2.23	[43]
Heart failure	1.00	1.20	1.70	1.00	1.20	1.20	[47]
Haemorrhagic stroke	1.27	2.19	2.38	NA	NA	NA	[45]
Oesophageal varices	1.26	9.54	9.54	1.26	9.54	9.54	[43]
Acute pancreatitis	1.30	1.80	3.20	1.30	1.80	1.80	[43]
Psoriasis	1.58	1.60	2.20	1.58	1.60	2.20	[43]

Males: Cat. I, 0–39.9 g/day, Cat. II, 40–59.9 g/day, Cat. III, ≥60 g/day

Females: Cat. I, 0–19.9 g/day, Cat. II, 20–39.9 g/day, Cat. III, ≥40 g/day

*NA* not applicable

**Table 3:** Age- and sex-specific alcohol-attributable fractions

Disease	Males					Females				
	6–19y	20–39y	40–59y	60–79y	≥80y	6–19y	20–39y	40–59y	60–79y	≥80y
Acute pancreatitis	0.20	0.23	0.26	0.26	0.19	0.20	0.20	0.22	0.20	0.15
Breast cancer	NA	NA	NA	NA	NA	0.11	0.11	0.12	0.11	0.08
Cardiac dysrhythmia	0.28	0.32	0.34	0.33	0.27	0.29	0.29	0.32	0.30	0.22
Haemorrhagic stroke	0.18	0.21	0.24	0.24	0.17	NA	NA	NA	NA	NA
Heart failure	0.01	0.01	0.03	0.04	0.01	0.00	0.00	0.01	0.01	0.01
Hypertension	0.25	0.28	0.32	0.33	0.24	0.25	0.24	0.27	0.25	0.19
Laryngeal cancer	0.40	0.44	0.48	0.48	0.39	0.41	0.41	0.46	0.43	0.34
Lip, pharynx cancer	0.28	0.31	0.36	0.36	0.26	0.28	0.27	0.32	0.28	0.21
Liver cancer	0.28	0.31	0.35	0.35	0.26	0.28	0.28	0.33	0.30	0.22
Oesophageal cancer	NA	0.42	0.45	0.45	0.37	NA	0.39	0.43	0.40	0.31
Oesophageal varices	0.29	0.31	0.44	0.47	0.24	0.29	0.27	0.45	0.38	0.28
Pancreatic cancer	0.08	0.09	0.10	0.11	0.07	0.08	0.08	0.10	0.08	0.06
Psoriasis	0.30	0.34	0.35	0.35	0.30	0.31	0.31	0.33	0.31	0.24
Rectal cancer	NA	0.07	0.09	0.09	0.06	NA	0.00	0.01	0.01	0.00
Unip major depression	0.14	0.16	0.20	0.20	0.13	0.36	0.36	0.42	0.38	0.30
Epilepsy	0.24	0.26	0.38	0.40	0.20	0.29	0.28	0.42	0.36	0.26
Chronic pancreatitis	0.20	0.23	0.26	0.26	0.19	0.20	0.20	0.22	0.20	0.15

*NA* not applicable; *Y* years

### Direct costs

#### Inpatient care

Costs associated with general hospital admissions were calculated by multiplying age- and sex-specific AAFs by the number of age- and sex-specific hospital care episodes/disease [17] and the corresponding unit cost [18] (Table 4).

**Table 4:** Disease-specific unit costs (€) used for the estimation of alcohol-attributable general hospital costs

Disease	Unit cost (€)	Source
Acute pancreatitis	5698	[17, 18]
Alcohol abuse	4190	[17, 18]
Alcoholic cardiomyopathy	5714	[17, 18]
Alcoholic dependence syndrome	4251	[17, 18]
Alcoholic gastritis	4607	[17, 18]
Alcoholic liver disease/cirrhosis	8729	[17, 18]
Alcoholic polyneuropathy	5538	[17, 18]
Alcoholic psychosis	4246	[17, 18]
Breast cancer	4941	[17, 18]
Cardiac dysrhythmia	4883	[17, 18]
Degeneration of nervous system due to alcohol	9250	[17, 18]
Foetal alcohol syndrome	2070	[17, 18]
Haemorrhagic stroke	7369	[17, 18]
Heart failure	7261	[17, 18]
Hypertension	5918	[17, 18]
Laryngeal cancer	6880	[17, 18]
Lip, oral cavity, pharynx cancer	6832	[17, 18]
Liver cancer	9403	[17, 18]
Oesophageal cancer	7414	[17, 18]
Oesophageal varices	6614	[17, 18]
Pancreatic cancer	7601	[17, 18]
Psoriasis	5814	[17, 18]
Rectal cancer	7748	[17, 18]
Unipolar major depression	7349	[17, 18]
Epilepsy	3405	[17, 18]

For non-surgical hospital day care, the unit cost was calculated in a different way, since its financing is not based on an average cost/disease, but on 11 groups of lump sums related to activities (for example infiltrations, medical imaging, biopsy) performed in non-surgical hospital day care [19]. An unweighted average cost was estimated based on the different lump sums. For psychiatric hospital care episodes, the number of age- and sex-specific alcohol-attributable care episodes [20] were multiplied by the corresponding mean length of stay and a fixed mean day price of €281.43 [21]. Pharmaceutical costs were not included since this information was not available. Estimation of the alcohol-attributable costs for sheltered housing and psychiatric nursing homes was made by multiplying the 2012 expenditures [22] by the proportion of alcohol-attributable admissions to those facilities being 13.4% and 8.1% for sheltered housing and psychiatric nursing homes, respectively [20]. Alcohol-attributable costs for inpatient rehabilitation and projects were available. The latter included two pilot projects of the Federal Public Service Health and three of the Federal Addiction Fund [22].

#### Outpatient care

No information of ambulatory alcohol-attributable physician contacts (general practitioners [GP] and medical specialists) was available. As a proxy, the 2012 expenditures for ambulatory physician contacts were multiplied by the proportion GPs (37.9%) and medical specialists (33.6%) [22]. A weighted average AAF, based on the age- and sex-specific AAFs applied for the general hospital costs was used to allow for an estimation of the costs that could be attributed to alcohol. For home-based nursing care, we first calculated the proportion of alcohol-attributable hospital admissions (*n* = 44,254) to the total number of hospital admissions in Belgium in 2012 (*n* = 1,667,051) [18]. This proportion was taken into account to the total expenditures for home-based nursing care [22]. For social work, other ambulatory services and two projects established by the Federal Addiction Fund, alcohol-attributable costs were available [22].

#### Pharmaceuticals

Data on prescribed pharmaceuticals used in alcohol disorders (acamprosate and disulfiram) were retrieved from the national drug consumption database [23]. The costs are restricted to prescribed and reimbursed drugs sold in pharmacies.

#### Prevention

Prevention costs included those of the association of GPs and the costs of specific alcohol prevention projects (for example the Federal Addiction Fund, the Flemish action plan tobacco, alcohol and drugs) [22].

### Indirect costs

Indirect costs included productivity losses from paid work due to disability (short-term and long-term) and premature mortality. Estimation of the costs occurred using the human capital approach measuring current and future productivity losses due to alcohol use in the reference year 2012 [5].

#### Disability

National disability statistics on the number of disabled people for the year 2012 were used [22]. This database contains sex-specific information on the number of disabled according to different disease groups (in accordance with major ICD-9 categories). In the current study, disease groups including diseases known to be associated with alcohol (neoplasms, mental disorders, nervous system diseases, circulatory diseases, digestive diseases, and skin diseases) were considered. For mental disorders, the proportion of people using psychiatric care due to alcohol (16.9%) [20] was used as a proxy to estimate the number of alcohol-attributable disabled. For the other disease groups, estimation of the proportion of alcohol-attributable disabled occurred by applying average AAFs derived from those used for the estimation of the general hospital costs. So, disability costs were estimated by multiplying the number of alcohol-attributable disabled individuals (5921 males; 7418 females) by the corresponding disability benefits (males, €46.32/day; females, €39.04/day) [22] and the mean number of disability days/disabled (*n* = 300) for the year 2012 [22].

#### Premature mortality

Estimation of productivity losses due to premature mortality was based on national mortality data including information on the number of deaths for different causes (by ICD-10 diagnosis) for different ages [24]. For the reference year 2012, disease-specific AAFs were multiplied by the number of deaths/alcohol-attributable disease, and a labour cost of €24,578 accounting for an employment rate of 61.8% [25]. The labour cost represents half of the annual labour cost being €49,156 since it was assumed that some premature mortality cases occurred at the beginning of the year and others at the end. For the following years, for each alcohol-attributable disease, disease-specific AAFs were multiplied by the number of potential productive life years lost (calculated by subtracting age at death from retirement age at 65 years) and multiplied by an annual labour cost of €49,156 [25].

### Intangible costs

Non-financial welfare costs were determined using disability-adjusted life years (DALYs). DALYs are a measure to quantify disease burden taking into account losses of healthy life years because of living with a disease and the years of life lost due to premature death [26]. Data on age- and sex-specific DALYs for Belgium for 2012 were available [27]. Again, AAFs were used to quantify the number of alcohol-attributable DALYs/disease. Monetary valuation of the DALYs occurred by applying a valuation of €40,000/DALY as suggested by Desaigues et al. [28] for European countries.

### Sensitivity analysis and scenario analysis

Social cost studies are likely to be characterized by some degree of uncertainty related to input parameters. Sensitivity and scenario analyses were applied to address uncertainties [26]. In the one-way sensitivity analysis, relative risk data were varied to assess which diseases had most influence on the costs. A probabilistic sensitivity analysis, based on 5000 simulations, was performed to assess the uncertainty in the key input parameters ‘relative risks’ and ‘unit costs’ by varying them concurrently. The cost categories related to psychiatric care, specific projects, prevention, rehabilitation, and ambulatory services were not included in the probabilistic sensitivity analysis, since for the estimation of these costs no relative risks were applied. Cost data were assumed to follow a gamma distribution and relative risks a lognormal distribution [29]. Scenario analysis made it possible to assess alternative modelling assumptions. In this analysis, the effects on the cost outcomes were evaluated taking into account a 10% decrease in alcohol consumption prevalence rates that were used to estimate AAFs. So, this analysis could only be performed for cost items for which AAFs were applied.

## Results

The health-related alcohol-attributable direct and indirect costs amounted to €906.1 million and €642.6 million, respectively (Table 5). This represents 0.4% of the gross domestic product (GDP) in Belgium in 2012. The majority of the direct costs were attributable to hospitalisation accounting for 82% of these costs (general hospitals: €245.7 million, 27.1%; psychiatric hospitals: €498.0 million, 55.0%) (Table 5). For those direct cost categories for whom sex-specific costs could be estimated, the majority were incurred by men (ranging from 62.1% to 81.6%) (Table 5). Calculation of the alcohol-attributable hospitalisation costs was based on 44,254 and 19,067 care episodes in general and psychiatric hospitals, respectively. This accounts for 2.7% and 13.2% of the total number of hospital care episodes in Belgium in 2012. The main cost drivers in general hospitals were circulatory diseases (35.7%), followed by mental disorders (24.3%) (Table 6). For each disease (except ‘skin diseases’), males were responsible for the largest share of the costs (Table 6). Alcohol-attributable physician contacts accounted for 11.4% (€103.6 million) of the direct costs, while the other categories each contributed less than 4%. Only 0.1% (€0.5 million) was spent on prevention (Table 5). The main cost driver within the indirect costs was premature mortality (€397.2 million, 62%) (Table 5). The number of life years lost up to the age of 65 years amounted to 7672. Males were responsible for 46.9% of the disability-associated costs (Table 5). Alcohol use was associated with an estimated 157,700 DALYs. This accounted for a total of €6,3 billion alcohol-attributable intangible costs.

**Table 5:** Total and sex-specific alcohol-attributable direct and indirect costs (million €) in Belgium, 2012

Cost category	Total costs	Percent	Sex-specific costs		% of costs by males
			Males	Females	
Direct costs	906,1				
Inpatient care					
General hospitals	245,7	27.1	155,7	90,0	63.4
Psychiatric hospitals	498,0	55.0	324,7	173,3	65.2
Sheltered housing	3,8	0.4	3,1	0,7	81.6
Psychiatric nursing homes	5,5	0.6	4,2	1,3	76.4
Rehabilitation	8,5	0.9	^a^	^a^	^a^
Specific projects	2,3	0.3	^a^	^a^	^a^
Outpatient care					
Physician contacts	103,6	11.4	64,6	39,0	62.4
Home-based nursing care	29,8	3.3	18,5	11,3	62.1
Ambulatory services	6,1	0.7	^a^	^a^	^a^
Specific projects	0,4	0.0	^a^	^a^	^a^
Pharmaceuticals	2,0	0.2	^a^	^a^	^a^
Prevention	0,5	0.1	^a^	^a^	^a^
Indirect costs	642,6				
Disability	245,4	38.2	115,2	130,2	46.9
Premature mortality	397,2	61.8	^a^	^a^	^a^

^a^no calculation of sex-specific costs possible due to restrictions in data availability

**Table 6:** General hospital alcohol-attributable costs (million €) according to disease groups

Disease group	Total costs	Percent	Sex-specific costs		% of costs by males
			Males	Females	
Circulatory diseases	87,8	35.7	60,0	27,8	68.3
Mental disorders	59,7	24.3	36,2	23,5	60.6
Digestive diseases	53,9	21.9	34,7	19,2	64.4
Neoplasms	28,9	11.8	16,3	12,6	56.4
Diseases of nervous system	15,0	6.1	8,2	6,8	54.4
Skin diseases	0,4	0.2	0,2	0,2	50.0
Total	245,7	100.0	155,6	90,1	

Sensitivity analysis and scenario analysis.

**Fig. 1:**
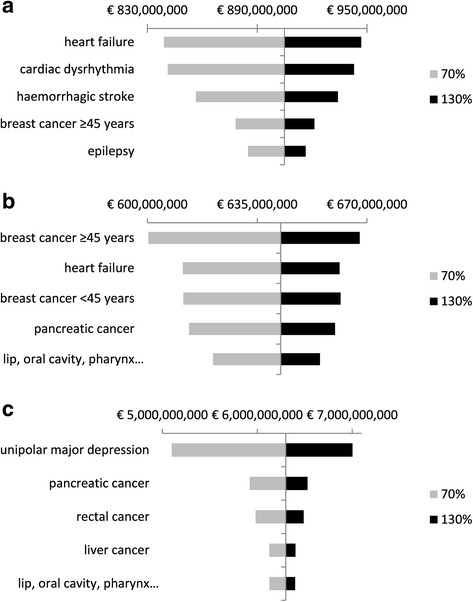
**a** One-way sensitivity analysis: effects on the direct costs varying the relative risks of diseases with 30% (top 5 diseases). **b** One-way sensitivity analysis: effects on the indirect costs varying the relative risks of diseases with 30% (top 5 diseases). **c** One-way sensitivity analysis: effects on the intangible costs varying the relative risks of diseases with 30% (top 5 diseases)

For the direct costs, the results reveal that the outcomes were most sensitive to a number of circulatory diseases (Fig. 1a). For the indirect costs, breast cancer ≥45 years was found to be most influencing the cost outcomes (Fig. 1b), while for the intangible costs this was unipolar major depression (Fig. 1c). The probabilistic sensitivity analysis resulted in an average cost for the direct cost categories ‘general hospitals’ of €246,2 million (95% CI: €215,9–€276,8 million), ‘physician contacts’ €104,3 million (95% CI: €92,5–€116,3 million), and ‘home-based nursing care’ €29,9 million (95% CI: €26,7–€33,1 million). For the indirect costs, an average cost of €245,7 million (95% CI: €239,3–€251,7 million) and €398,0 million (95% CI: €371,1–€424,6 million) was found for ‘disability’ and ‘premature mortality’, respectively. For the intangible costs, an average cost of €6.3 billion (95% CI: €5.8–€6.8 billion) was observed. Assuming a 10% decrease of alcohol consumption would result in €65,5 million (7.2%) direct cost savings and €20,6 million (3.2%) indirect cost savings (Table 7).

**Table 7:** Scenario analysis: effects on the costs (€) assuming a 10% decrease of alcohol consumption

Cost category	Base case	10% decrease	∆ cost	% change
Direct costs				
Inpatient care				
General hospitals	245,744,850	234,924,244	-10,820,607	4.4
Psychiatric hospitals	498,032,609	448,229,348	−49,803,261	10.0
Sheltered housing	3,814,635	3,684,534	−130,101	3.4
Psychiatric nursing homes	5,483,377	5,322,647	−160,730	2.9
Outpatient care				
Physician contacts	103,560,056	101,024,602	−2,535,454	2.5
Home-based nursing care	48,246,624	46,171,799	−2,074,825	4.3
Total	904,882,152	839,357,174	−65,524,978	7.2
Indirect costs				
Disability	245,368,931	236,464,785	−8,904,146	3.6
Premature mortality	397,156,108	385,427,049	−11,729,059	3.0
Total	642,525,039	621,891,835	−20,633,205	3.2
Intangible costs				
Total	6,300,017,986	6,098,578,249	−201,439,736	3.2

## Discussion

The alcohol-attributable direct and indirect costs were estimated at €906,1 million and €642,6 million, respectively. Alcohol resulted in 157,700 DALYs accounting for an estimated €6,3 billion non-financial welfare costs. The direct and indirect alcohol-attributable health costs represented 0.4% of the GDP. By presenting the findings in this way, they could be compared with those from social cost studies from other countries. For example, in a social cost study of the economic impact of alcohol in Germany, alcohol-attributable costs accounted for 1.16% of the GDP. In this study, productivity losses from unpaid work were included. Without considering these costs, the total costs accounted for 0.89% of the GDP [8]. In Sweden, alcohol-attributable costs were found to represent 0.9% of the GDP [9]. In the latter study, cost estimates also included crime costs. In the current study, the main cost driver in the direct cost category were the costs associated with inpatient hospital care. This is different from the results of previous studies on the economic impact of alcohol to society. For example, the results of a social cost study in Sweden identified the social service costs as the most important cost category, followed by crime costs and inpatient care costs. This was explained by the fact that a substantial part of the treatment of individuals suffering from alcohol problems is performed within social welfare services [9]. Similar results were found in a social cost study of alcohol in Scotland. In that study, alcohol-attributable hospitalisation costs were preceded by crime costs and social work services costs [7]. Comparing the results of social cost studies is difficult and must be cautiously interpreted due to differences in methodologies such as the types of cost items included or calculation methods used [30]. Nevertheless, even if a uniform methodology was to be developed and used, a cross-country comparison would necessitate sufficient contextualisation, since countries differ in terms of social security systems, institutional structures, cultural traditions.

The majority of the direct costs were associated with hospitalisation. To decrease the number of alcohol-attributable hospitalisations, the role of ambulatory health care services in the detection, diagnosis, early intervention, and care for people with alcohol problems should be strengthened. According to the findings of a study on primary care in 31 European countries, a strong primary care is associated with better population health and lower unnecessary hospital admissions [31]. Effective primary care can improve health and prevent diseases at earlier stages, but also stimulates people to engage in healthier behaviours [32]. Alcohol was also associated with considerable costs related to productivity losses. In Belgium, many companies are unaware of the impact of substance misuse or underestimate this problem [33]. So, workplace interventions may be useful and the occupational physician might play an important role in the field of substance misuse as health and safety promotor. For the estimation of the disability costs due to premature death, life years lost were not discounted. Our study can be considered as a budget impact analysis. According to guidelines from the International Society for Pharmacoeconomics and Outcomes Research, discounting is not recommended [34]. Contrary to previous studies examining the social costs of alcohol, intangible costs were also estimated. It is yet difficult to place a monetary value upon welfare losses resulting in the fact that intangible costs are often ignored in social cost studies of substance misuse [35]. In fact, in some previous studies examining the economic burden of alcohol, welfare losses were not included into the analysis at all [7, 14], while in other studies, welfare losses were reported as losses of quality-adjusted life years, but not valued in monetary terms [9, 13]. By including the intangible costs, we were able to examine the impact of alcohol on quality of life in monetary terms. The current study can be considered as the most comprehensive analysis of the costs associated with alcohol use. One could argue that there may be a double counting between the indirect costs and the intangible costs. Following the guideline ‘Best practices of estimating the costs of alcohol’ [6], we considered them as separate cost categories. Only estimating the alcohol-attributable direct and indirect costs would have resulted in omitting considerable aspects of the total alcohol-attributable burden [26]. The results revealed that only a small amount of the expenditures (0.1% of direct costs) was spent on prevention. The importance of lowering the burden of alcohol was showed in the scenario analysis identifying savings of about €65.5 million direct costs and €20.6 million indirect costs. There is evidence supporting the efficiency of particular strategies aimed at preventing or reducing the burden of alcohol. For example, the World Health Organization identifies a number of ‘best buy’ interventions including tax increases, restricted access to retailed alcohol, and enforcing bans on alcohol advertising [36]. Alcohol use does not only cause costs to society, but is also associated with financial benefits. The contribution of the alcohol industry could be considered as such a benefit [37]. For example, the alcohol production and consumption helps the economy by increasing employment, tax revenues and technology transfers [38].

A number of limitations need to be addressed. First, for a number of cost categories, no alcohol-attributable costs could be estimated due to the absence of (reliable) data for Belgium. These categories (ambulatory Accident and Emergency Department visits, non-medical home-based care, pharmaceutical use in inpatient psychiatric facilities, and lost productivity due to presentism and unpaid work) were not included in the analysis. It is important to provide information on these cost categories, since the aim of a social cost study is not only to examine the economic impact of a disease or condition, but also to identify data gaps and desirable refinements of (national) registration systems [39]. By reporting our ‘missing cost categories’, it was the aim to provide a more comprehensive overview of relevant cost categories for a social cost study of alcohol. This may serve as a guiding tool for future such studies. For other cost categories (for example home-based nursing care, ambulatory physician visits), assumptions and extrapolations were applied to provide an estimation of the costs. It is clear that the development of more accurate data collection systems should receive priority, so that they can serve as reliable information sources facilitating the calculation of more robust cost estimates. Second, alcohol consumption data may be characterized by a certain level of uncertainty, since consumption data were based on self-reported data. It is known that such data tend to underestimate the real amount of substance use [40]. Third, for certain diseases (for example colon cancer, chronic pancreatitis, short gestation/low birthweight), no relative risks were found and as a consequence, those diseases were not accounted for in the analysis. Fourth, for a number of cost categories (for example physician contacts, home-based nursing care, indirect costs, intangible costs), no information on the proportion of cases that could be attributed to alcohol was available. To enable an estimation of the alcohol-attributable costs for these cost categories, AAFs were estimated based on those used in the calculation process for the general hospitals. By applying this method, we assumed similar patterns of relative weights of diseases treated in hospitals as in the cost categories with no specific AAFs. It is yet unlikely that, for example, patterns or proportions of diseases treated in hospitals are similar to those seen by GPs. These AAFs are thus likely characterized by some degree of uncertainty. Similar problems were found in previous research. For example, Neubauer et al. [41] estimated the direct and indirect tobacco-attributable costs in Germany. In that study, the substance-attributable fraction for mortality was used as a proxy for the estimation of tobacco-associated health care use and expenditures. Fifth, interaction effects of combined use of alcohol and other substances (for example illicit drugs or psychoactive pharmaceuticals) on the risk of developing alcohol-attributable diseases were not accounted for. This may have resulted in an underestimation of the costs, since relative risks for certain diseases may be higher in multiple substance users. For example, the combined use of opioid pain relievers, benzodiazepines and alcohol is likely to result in an increased risk of adverse events [42]. It is clear that the findings are affected by the limitations and may have resulted in an underestimation of the reality. Social cost studies are frequently characterized by methodological dilemmas and issues [5]. Therefore, the results should be carefully interpreted and considered as ‘estimates’. The uncertainty of the cost estimates was addressed in the sensitivity analyses. Uncertainty in cost-of-illness studies is almost self-evident. It is however necessary to address these issues and to inform the reader about the amount of uncertainty associated with the estimate [5]. In the current study, varying the relative risk data of the diseases known to be associated with alcohol revealed that the direct health-related costs were most sensitive to a number of circulatory diseases, while for the indirect costs this was breast cancer. A uniform 70% to 130% uncertainty was applied in the one-way sensitivity analysis, since this analysis was conducted to gain insight in the parameters most influencing the study outcome. The full uncertainty around key input parameters was reflected with the probabilistic sensitivity analysis. Insights into the economic impact of alcohol as well as other substances including tobacco, illicit drugs and psychoactive pharmaceuticals are important to strengthen the knowledge of the current burden due to substance use. The study findings help policy makers to understand the scale of problems issuing from alcohol use and to target specific concerns and policies. For example, the study identified different alcohol-attributable cost categories confirming that the substance phenomenon is multidimensional. Various health and welfare services are confronted with the problem of alcohol. So, the results can allow decision makers to monitor the resource allocation in accordance with the economic burden of the different health problems. It is yet clear that evidence-based policy making needs comprehensive information which can only be completed by combining information of social costs together with other sources of information such as data about new trends in substance use, data about specific target groups related to prevention. In addition, the information from social cost studies may serve as input to determine the efficiency of interventions aimed at reducing the burden of alcohol and other substances, assisting governments in setting priorities in their healthcare policies. This necessitates the development of social cost studies based on sound methodological principles.

## Conclusion

The results of the study demonstrate the substantial economic impact of alcohol to the Belgian society and can be considered as the most comprehensive analysis of the alcohol-attributable costs in Belgium so far. Future studies examining the social costs of alcohol or other addictive substances should ideally deal with the limitations identified in the current study. Further, social cost studies should include subgroup analyses to assess the impact of socioeconomic variables on cost outcomes. By doing that, it will be possible to formulate specific policy recommendations for different target groups. In the current study, subgroup analysis was not possible due to limitations in availability and accessibility of data necessary for such analyses. This necessitates the development of more accurate data collection systems so that they can serve as reliable information sources facilitating the calculation of more robust cost estimates. The study estimated the resources that alcohol makes unavailable for other purposes. From an economic policy perspective, it may also be relevant to determine the feasible minimum cost, i.e. the fraction of total attributable costs that may potentially be averted by reducing exposure of alcohol (mis)use by policy interventions.

## Abbreviations

AAF: Alcohol-attributable fraction
CI: Confidence interval
DALY: Disability-adjusted life year
GDP: Gross domestic product
GP: General practitioner
HRQOL: Health-related quality of life
ICD: International Classification of Diseases
NA: Not applicable
RR: Relative risk

## References

[1] World Health Organization (2014). Global status report on alcohol and health 2014.

[2] Rehm J, Mathers C, Popova S, Thavorncharoensap M, Teerawattananon Y, Patra J (2009). Global burden of disease and injury and economic cost attributable to alcohol use and alcohol-use disorders. Lancet.

[3] Thavorncharoensap M, Teerawattananon Y, Yothasamut J, Lertpitakpong C, Chaikledkaew U (2009). The economic impact of alcohol consumption: a systematic review. Subst Abuse Treat Prev Policy.

[4] Rice DP (2000). Cost of illness studies: what is good about them?. Inj Prev.

[5] Moore TJ, Caulkins JP (2006). How cost-of-illness studies can be made more useful for illicit drug policy analysis. Appl Health Econ Health Policy.

[6] Moller L, Matic S (2010). Best practices of estimating the costs of alcohol - recommendations for future studies.

[7] Varney SJ, Guest JF (2002). The annual societal cost of alcohol misuse in Scotland. PharmacoEconomics.

[8] Konnopka A, Konig HH (2007). Direct and indirect costs attributable to alcohol consumption in Germany. PharmacoEconomics.

[9] Jarl J, Johansson P, Eriksson A, Eriksson M, Gerdtham UG, Hemstrom O, Selin KH, Lenke L, Ramstedt M, Room R (2008). The societal cost of alcohol consumption: an estimation of the economic and human cost including health effects in Sweden, 2002. Eur J Health Econ.

[10] Vander Laenen F, De Ruyver B, Christiaens J, Lievens D (2011). Drugs in Figures III.

[11] Degreef T, Pacolet J, Bouten R (2003). Social cost-benefit analysis of alcohol use and misuse in Belgium.

[12] Single E, Collins D, Easton B, Harwood H, Lapsley H, Kopp P, Wilson E (2003). International guidelines for estimating the costs of substance abuse - second edition.

[13] Konnopka A, Konig HH (2009). The health and economic consequences of moderate alcohol consumption in Germany 2002. Value Health.

[14] Rehm J, Gnam W, Popova S, Baliunas D, Brochu S, Fischer B, Patra J, Sarnocinska-Hart A, Taylor B (2007). The costs of alcohol, illegal drugs, and tobacco in Canada, 2002. J Stud Alcohol Drugs.

[15] Kleinbaum D, Kupper L, Morgenstern H (1982). Epidemiologic research, principles and quantitative methods.

[16] Health Interview Survey 2013 [https://hisia.wiv-isp.be/SitePages/Home.aspx].

[17] Federal Public Service Health. Minimum Hospital Data [https://www.health.belgium.be/nl/gezondheid/organisatie-van-de-gezondheidszorg/ziekenhuizen/registratiesystemen/mzg].

[18] Federal Public Service Health, National Institute for Health and Disability Insurance. National database medical diagnosis/care and cost. [https://tct.fgov.be/webetct/etct-web/html/nl/index.jsp].

[19] Van de Sande S, Swartenbroekx N, Van de Voorde C, Devos C, Devriese S (2012). Evolution of day-care: impact of financing and regulation. Health services research (HSR). KCE reports 192A. D/2012/10.273/89.

[20] Federal Public Service Health. Minimum psychiatric data [https://www.health.belgium.be/nl/gezondheid/organisatie-van-de-gezondheidszorg/ziekenhuizen/registratiesystemen/mpg].

[21] National Institute for Health and Disability Insurance. Hospital day prices [http://www.riziv.fgov.be/nl/themas/kost-terugbetaling/door-ziekenfonds/verzorging-ziekenhuizen/Paginas/verpleegdagprijzen-ziekenhuizen.aspx#.WeYVWGdsTT8].

[22] National Institute for Health and Disability Insurance. Annual Report 2013 [http://www.riziv.fgov.be/SiteCollectionDocuments/jaarverslag-2013.pdf].

[23] National Institute for Health and Disability Insurance. Statistics on pharmaceuticals delivered in public pharmacies (Farmanet) [http://www.riziv.fgov.be/nl/statistieken/geneesmiddel/Paginas/Statistieken-geneesmiddelen-apotheken-farmanet.aspx#.WeYUZmdsTT8].

[24] Federal Public Service Economy. Population - causes of death 1998–2014 [http://statbel.fgov.be/nl/modules/publications/statistiques/bevolking/downloads/bevolking_-_doodsoorzaken.jsp#.WeXp7GdsTT8].

[25] Federal Public Service Economy. Analysis - Belgian labour market trends (1983–2014) [http://statbel.fgov.be/nl/modules/publications/statistiques/arbeidsmarkt_levensomstandigheden/analyse_-_tendances_sur_le_marche_du_travail_belge_1983-2013_.jsp].

[26] Drummond M, Sculpher M, Torrance G, O'Brien B, Stoddart G (2005). Methods for the economic evaluation of health care programmes.

[27] World Health Organization. Estimated DALYs by cause, sex and WHO member state. [http://www.who.int/healthinfo/global_burden_disease/estimates/en/index2.html].

[28] Desaigues B, Ami D, Hutchison M, Chilton S, Metcalf H, Hunt A, Ortiz R, Navrud S, Kaderjak P, Szanto R (2006). Final report on the monetary valuation of mortality and morbidity risks from air pollution.

[29] Briggs A, Claxton K, Sculpher M (2006). Decision modelling for health economic evaluation.

[30] World Health Organization (2009). WHO guide to identifying the economic consequences of disease and injury.

[31] Kringos DS, Boerma W, van der Zee J, Groenewegen P (2013). Europe's strong primary care systems are linked to better population health but also to higher health spending. Health Aff (Millwood).

[32] EXPH (2014). EXpert panel on effective ways of investing in health. Report on definition of a frame of reference in relation to primary care with a special emphasis on financing systems and referral systems.

[33] Tecco J, Jacques D, Annemans L (2013). The cost of alcohol in the workplace in Belgium. Psychiatr Danub.

[34] Sullivan SD, Mauskopf JA, Augustovski F, Jaime Caro J, Lee KM, Minchin M, Orlewska E, Penna P, Rodriguez Barrios JM, Shau WY (2014). Budget impact analysis-principles of good practice: report of the ISPOR 2012 budget impact analysis good practice II task force. Value Health.

[35] Single E (2003). Estimating the costs of substance abuse: implications to the estimation of the costs and benefits of gambling. J Gambl Stud.

[36] World Health Organization (2011). Discussion paper: prevention and control of non-communicable diseases: priorities for investment.

[37] Horlings E, Scoggins A (2006). An ex ante assessment of the economic impacts of EU alcohol policies.

[38] Claeson M (2000). World Bank Group note on alcohol beverages.

[39] Single E (2009). Why we should still estimate the costs of substance abuse even if we needn't pay undue attention to the bottom line. Drug Alcohol Rev.

[40] Chick J, Kemppainen E (2007). Estimating alcohol consumption. Pancreatology.

[41] Neubauer S, Welte R, Beiche A, Koenig HH, Buesch K, Leidl R (2006). Mortality, morbidity and costs attributable to smoking in Germany: update and a 10-year comparison. Tob Control.

[42] Ogbu UC, Lotfipour S, Chakravarthy B (2015). Polysubstance abuse: alcohol, opioids and benzodiazepines require coordinated engagement by society, patients, and physicians. West J Emerg Med.

[43] Rehm J, Room R, Monteiro M, Gmel G, Graham K, Rehn N, Sempos CT, Frick U, Jernigan D, Ezzati M, Lopez A, Rodgers A, Murray C (2003). Comparative quantification of health risks. Global and regional burden of disease attributable to selected major risk factors.

[44] Rehm J, Sulkowska U, Manczuk M, Boffetta P, Powles J, Popova S, Zatonski W (2007). Alcohol accounts for a high proportion of premature mortality in central and eastern Europe. Int J Epidemiol.

[45] Rehm J, Room R, Graham K, Monteiro M, Gmel G, Sempos CT (2003). The relationship of average volume of alcohol consumption and patterns of drinking to burden of disease: an overview. Addiction.

[46] Gilman SE, Abraham HD (2001). A longitudinal study of the order of onset of alcohol dependence and major depression. Drug Alcohol Depend.

[47] Klatsky AL, Chartier D, Udaltsova N, Gronningen S, Brar S, Friedman GD, Lundstrom RJ (2005). Alcohol drinking and risk of hospitalization for heart failure with and without associated coronary artery disease. Am J Cardiol.

